# Cell-Based Drug Delivery Platforms

**DOI:** 10.3390/pharmaceutics13010002

**Published:** 2020-12-22

**Authors:** José M. Lanao, Carmen Gutiérrez-Millán, Clara I. Colino

**Affiliations:** 1 Area of Pharmacy and Pharmaceutical Technology, Department of Pharmaceutical Sciences, University of Salamanca, 37007 Salamanca, Spain; jmlanao@usal.es (J.M.L.); ganda@usal.es (C.I.C.); 2 The Institute for Biomedical Research of Salamanca (IBSAL), 37007 Salamanca, Spain

Within the framework of nanomedicine, drug delivery has experienced rapid progress in recent years. Delivery systems based on cells or their derivative products such as erythrocytes, platelets, stem cells, dendritic cells, or extracellular vesicles may be used as drug delivery platforms and are interesting and promising alternatives to other nanosystems, such as nanoparticles. These systems can be applied in diverse therapeutic fields, such as cancer, cardiovascular therapies, anti-infective treatments, or vaccines. The purpose of this Special Issue is to present the most recent research in the field of delivery systems based on cells, modified cells, and derivative systems, and their therapeutic applications. Four research articles and eight review articles are published in this Special Issue.

The use of stem cells for drug delivery constitutes a promising strategy with potential applications in the fields of cancer therapy, regenerative medicine, and immunotherapy. Three papers about these topics are included in this Special Issue.

The manuscript of Borghese C et al. addresses the use of adipose-derived stem cells (ADSCs) as a drug delivery system of paclitaxel (PTX) against ovarian cancer. The anticancer activity of paclitaxel was evaluated in ovarian cancer cells sensitive or resistant to paclitaxel using two-dimensional (2D) and three-dimensional (3D) (heterospherical) in vitro models. The results of this study demonstrated that PTX-ADSCs inhibited cell migration and dissemination in ovarian cancer and overcame the intrinsic PTX resistance of Kuramochi cells and the extrinsic resistance induced by aggregation of tumor cells. This work demonstrates the potential utility of mesenchymal stem-cell-based chemotherapy drug delivery systems for cancer therapy [1].

The large-scale production of mesenchymal stromal cells (MSCs) loaded with paclitaxel under good manufacturing practices (GMP) for clinical applications in cancer therapeutics has also been suggested. A novel system has been developed based on a closed bioreactor system (quantum cell expansion system) that allows the production of MSCs loaded with chemotherapeutic agents such as paclitaxel on an industrial scale. In addition, it has been shown that cryopreservation maintains the viability of cells in terms of recovery and drug release capacity, which allows its allogeneic use for patient treatment over time [2].

In the field of immunotherapy, the manuscript of García-Pérez et al. addresses a review on the use of autologous hematopoietic stem cells for ex vivo gene therapy for immuno-deficiencies from pre-clinical stages to clinical trials. The work reviews aspects such as product development based on in vivo and ex vivo therapies, depending on target cells of interest, vector designs, ex vivo manipulation and transduction efficiency and the use of models to test the efficacy of gene therapy such as in vitro models or animal models. The safety and toxicology assessment and the scaling-up under good manufacturing practices (GMP) of gene therapy to obtain a product suitable for clinical use are also discussed in the manuscript [3].

Among the different cells that have been studied as platforms for drug delivery, red blood cells (RBC) and their derivatives undoubtedly stand out, and three papers of this Special Issue focus on them. Their physiological characteristics, ubiquitous presence, and great capacity to harbor different kinds of cargo make erythrocytes ideal candidates as vehicles of drugs or other molecules with therapeutic applications.

There are two main types of methods for the association of therapeutic molecules and RBCs—encapsulation inside of them or surface loading. Inclusion within erythrocytes is the most common approach, and can be achieved through different techniques, such as osmosis-based methods, electroporation, or induced endocytosis.

The paper of Koleva et al. included in this Special Issue compiles the applications in which, since the first studies in the 1970s, bioactive substances have been loaded inside erythrocytes. Erythrocytes have been used mainly for two different purposes—targeting drugs to specific organs, primarily those related to the reticuloendothelial system (RES), and acting as carriers from which a gradual release occurs. Loaded erythrocytes have been employed as bioreactors, for pharmacological agent release, in diagnostics or to express targeted therapeutic proteins after being genetically modified. This paper also includes an interesting overview of the main limitations of carrier erythrocytes and the companies that have effectively brought products based on this technology to clinical practice [4].

Encapsulation inside erythrocytes protects the cargo from exposure to blood components that can interact or even degrade the molecules until they reach their target. Nevertheless, other authors have also proposed the association of molecules onto the erythrocyte surface. Although an excessive modification of the erythrocyte’s native characteristics can be detrimental, surface association can offer other advantages such as the possibility of in vivo loading and the potential of direct interaction of active molecules with vascular endothelium. 

The article from Glassman et al. is focused on strategies based on surface coupling and the pharmacokinetic modifications induced by them. The coupled molecule is less accessible for interaction with medium components and this fact could even alter its pharmacological profile. This paper focuses mainly on research work employing erythrocyte surface-coupling strategies and the improvement in pharmacokinetics that can be achieved by them, as well as the specific unique pharmacokinetic properties resulting from the establishment of a dynamic system between cells, plasma, and tissues [5].

The third paper included in this Special Issue related to the carrier erythrocyte platform, by B.E. Bax, is focused on the association of erythrocytes with enzymes, a field in which carrier erythrocytes have been widely and successfully applied. Inclusion in erythrocytes is especially advantageous for enzymes since it provides them protection from degradation and immunological interactions. Applications of these systems have mainly been proposed for metabolic-disease enzyme replacement but also for antitumor therapy, thrombolytic therapy, and even for exogenous chemical detoxification. This article compiles the research work developed in this field as well as the limitations and challenges that must be overcome [6].

In addition to stem cells and erythrocytes, dendritic cells (DCs) also have a prominent role in the field of cell-based drug delivery. These antigen-presenting cells have mostly been applied in cancer therapy and autoimmune and inflammatory diseases.

The work of Koya et al. proposes a cancer vaccine based on DCs containing Wilms’ tumor (WT1) peptides, molecules that are expressed in various types of solid tumors. Their WT1-DCs join the innate adjuvant properties of DCs with the properties of these targeting tumor-associated antigens (TAAs), leading to low production of the immunosuppressive cytokine IL-10 and higher viability and recovery of DC/monocyte ratio than conventional DCs. Even more, DCs induced acquired immunity which was observed after their administration to patients with cancer, demonstrating their potential in clinics [7].

Other cells, not so thoroughly studied as the previous ones discussed, are also represented in this Special Issue.

Munteanu et al. collect the possibilities of adipocyte-based cell therapy in oncology in their paper. It has been demonstrated that adipose tissue not only plays an energy storage role, but it is interconnected with peripheral organs and tissues, is implicated in paracrine and endocrine signaling, and is involved, through different mechanisms, in the installation and development of pathological status, such as cancer.

This article reviews the main methods of adipose-cell isolation, loading, and characterization, as well as the applications of engineered adipose-tissue-derived cells in oncology, including mesenchymal stem cells derived from adipose tissue, conjugations with nanoformulations for drug delivery, and adipocyte-derived exosomes. The authors revise the studies found in literature up to the present and point out the future perspectives in this field [8].

Immunotherapy based on the use of chimeric antigen receptor (CAR)-T-cell therapy has emerged strongly among cellular therapies for hematological cancer treatments and is analyzed in the article by Roex et al. This strategy uses T cells that are extracted from the patient, transfected in vitro with RNA encoding the CAR by using viral vectors, expanded by incubation, and administrated to the patient by intravenous infusion. The target antigen most frequently chosen is CD19 and second generations of CAR-T cells include co-stimulatory domains such as CD20 or 4-1BB. At the moment, there are two therapies approved by the European Medicines Agency (EMA) and the Food and Drug administration (FDA) for treating certain B cell malignancies, and CAR-T cells are being studied for their use in other pathologies. The article included in this Special Issue revises CAR-T-cell preparation and administration, as well as the main findings about the efficacy and toxicity of CAR-T cells obtained in clinical trials for the principal hematological malignancies for which these therapies are intended. The main problems identified for this treatment are the durability of the effects and the toxicity. The strategies that are proposed to obtain durable responses, tackle relapses, and improve the safety profile are also discussed [9].

Extracellular vesicles (EVs) as biological carriers with therapeutic purposes are the focus of the work of Gangadaran et al. EVs comprise different kinds of small vesicles derived from cells that differ in their origin and size. In order to overcome the scarce yield of EVs obtained and increase the production efficiency, extracellular vesicle mimetics (EVMs) and mixed vesicles with liposomes have been developed. This work goes beyond the EVMs production processes of extrusion, sonication, freeze-thaw, and incubation. Also, the methods for drug loading in EVs and EVMs are discussed along with their advantages and disadvantages. The need to standardize the production and characterization of the vesicles is pointed out as a challenge to their clinical development [10].

In relation to EVs, Ragni and colleagues recognize them as a promising cell-based drug delivery platform for endogenously- or exogenously-loaded miRNA. According to the requirement for EV standardization in basic research and the therapeutic use of these vesicles, they tried to define universal miRNA for characterizing the potency of EVs from different batches or origins. They identified and characterized the stability of miRNA reference genes (RGs) in human amniotic membrane-derived mesenchymal stromal cells (hAMSC). They used four different applets to identify the most stable miRNA and found miR-101-30 and miR-22-5p to be the best performers in hAMSC-EVs and proposed miR-22-5p as an RG in extracellular vesicles obtained from other sources. Characterization of extracellular vesicles’ miRNA is also important when they are used as vehicles to treat related miRNA pathologies in order to select the best batch for each therapeutic application [11].

As a kind of extracellular vesicle, mesenchymal stem cell (MSC) exosomes have been attributed the anti-inflammatory and regenerative properties of MSC. Harrell et al. make a revision of these small vesicles generated from the latter endosomes. They go through their main components—lipids, proteins, and miRNA, as well as the molecular and cellular mechanisms responsible for their effects. MSC exosomes are able to inhibit the expression of inflammatory cytokines and induce the generation of the immunosuppressive and/or anti-inflammatory M2 phenotype in macrophages, microglial cells, and dendritic cells. The signaling cascades that protect parenchymal cells and promote their regeneration are also explained. Finally, the challenges to the clinical use of MSC exosomes are discussed and promising results from clinical trials for therapeutic conditions such as chronic kidney disease, ischemic stroke, and dry eye as well as different administrations routes are presented [12].

## References

[1] Borghese C., Casagrande N., Corona G., Aldinucci D. (2020). Adipose-Derived Stem Cells Primed with Paclitaxel Inhibit Ovarian Cancer Spheroid Growth and Overcome Paclitaxel Resistance. Pharmaceutics.

[2] Lisini D., Nava S., Frigerio S., Pogliani S., Maronati G., Marcianti A., Coccè V., Bondiolotti G., Cavicchini L., Paino F. (2020). Automated Large-Scale Production of Paclitaxel Loaded Mesenchymal Stromal Cells for Cell Therapy Applications. Pharmaceutics.

[3] Garcia-Perez L., Ordas A., Canté-Barrett K., Meij P., Pike-Overzet K., Lankester A., Staal F.J.T. (2020). Preclinical Development of Autologous Hematopoietic Stem Cell-Based Gene Therapy for Immune Deficiencies: A Journey from Mouse Cage to Bed Side. Pharmaceutics.

[4] Koleva L., Bovt E., Ataullakhanov F., Sinauridze E. (2020). Erythrocytes as Carriers: From Drug Delivery to Biosensors. Pharmaceutics.

[5] Glassman P.M., Villa C.H., Ukidve A., Zhao Z., Smith P., Mitragotri S., Russell A.J., Brenner J.S., Muzykantov V.R. (2020). Vascular Drug Delivery Using Carrier Red Blood Cells: Focus on RBC Surface Loading and Pharmacokinetics. Pharmaceutics.

[6] Bax B.E. (2020). Erythrocytes as Carriers of Therapeutic Enzymes. Pharmaceutics.

[7] Koya T., Date I., Kawaguchi H., Watanabe A., Sakamoto T., Togi M., Kato T., Yoshida K., Kojima S., Yanagisawa R. (2020). Dendritic Cells Pre-Pulsed with Wilms’ Tumor 1 in Optimized Culture for Cancer Vaccination. Pharmaceutics.

[8] Munteanu R., Onaciu A., Moldovan C., Zimta A.-A., Gulei D., Paradiso A.V., Lazar V., Berindan-Neagoe I. (2020). Adipocyte-Based Cell Therapy in Oncology: The Role of Cancer-Associated Adipocytes and Their Reinterpretation as Delivery Platforms. Pharmaceutics.

[9] Roex G., Feys T., Beguin Y., Kerre T., Poiré X., Lewalle P., Vandenberghe P., Bron D., Anguille S. (2020). Chimeric Antigen Receptor-T-Cell Therapy for B-Cell Hematological Malignancies: An Update of the Pivotal Clinical Trial Data. Pharmaceutics.

[10] Gangadaran P., Ahn B.-C. (2020). Extracellular Vesicle- and Extracellular Vesicle Mimetics-Based Drug Delivery Systems: New Perspectives, Challenges, and Clinical Developments. Pharmaceutics.

[11] Ragni E., Perucca Orfei C., Silini A.R., Colombini A., Viganò M., Parolini O., de Girolamo L. (2020). miRNA Reference Genes in Extracellular Vesicles Released from Amniotic Membrane-Derived Mesenchymal Stromal Cells. Pharmaceutics.

[12] Harrell C.R., Jovicic N., Djonov V., Volarevic V. (2020). Therapeutic Use of Mesenchymal Stem Cell-Derived Exosomes: From Basic Science to Clinics. Pharmaceutics.

